# Association between Cytomegalovirus Antibody Levels and Cognitive Functioning in Non-Elderly Adults

**DOI:** 10.1371/journal.pone.0095510

**Published:** 2014-05-20

**Authors:** Faith Dickerson, Cassie Stallings, Andrea Origoni, Emily Katsafanas, Lucy A. B. Schweinfurth, Christina L. G. Savage, Robert Yolken

**Affiliations:** 1 Stanley Research Program, Sheppard Pratt Health System, Baltimore, Maryland, United States of America; 2 Department of Pediatrics, Johns Hopkins School of Medicine, Baltimore, Maryland, United States of America; University of Regensburg, Germany

## Abstract

**Background:**

Elevated levels of antibodies to Cytomegalovirus (CMV) have been associated with cognitive impairment, but the quantitative relationship between CMV antibody levels and domains of cognitive functioning in younger adults has not been established.

**Methods:**

We measured IgG class antibodies to Cytomegalovirus in 521 individuals, mean age 32.8 years. Participants were selected for the absence of psychiatric disorder and of a serious medical condition that could affect brain functioning. Cognitive functioning was measured with the Repeatable Battery for the Assessment of Neuropsychological Status (RBANS), the Wisconsin Card Sorting Test, Trail Making Test part A, and the WAIS III Letter Number Sequencing subtest. Linear regression analyses were used to measure the quantitative association between cognitive scores and Cytomegalovirus IgG antibody level. Logistic regression analyses were used to measure the odds of low cognitive scores and elevated antibody levels defined as an antibody level > = 50^th^, 75^th^, and 90^th^ percentile of the group.

**Results:**

Higher levels of CMV antibodies were associated with lower performance on RBANS Total (coefficient −1.03, p<.0002), Delayed Memory (coefficient −0.94, p<.001), Visuospatial/Constructional (coefficient −1.77, p<5×10^−7^), and Letter Number Sequencing (coefficient −0.15, p<.03). There was an incremental relationship between the level of CMV antibody elevation and the odds of a low RBANS Total score. The odds of a low total cognitive score were 1.63 (95^th^ % CI 1.01, 2.64; p<.045), 2.22 (95^th^ % CI 1.33, 3.70; p<.002), and 2.46 (95^th^ % CI 1.24, 4.86; p<.010) with a CMV antibody level greater than or equal to the 50^th^, 75^th^, and 90^th^ percentile respectively.

**Conclusions:**

Higher levels of Cytomegalovirus antibodies are associated with lower levels of cognitive functioning in non-elderly adults. Methods for the prevention and treatment of CMV infection should be evaluated to determine if they result in an improvement in cognitive functioning in otherwise healthy adults.

## Introduction

Cytomegalovirus or CMV is a beta herpes virus which can infect the central nervous system of neonates and immune compromised individuals [1], [2] and, rarely, can cause encephalitis in immune competent individuals [3]. CMV establishes latency within myeloid progenitor cells of humans (Proceedings of the 11^th^ International CMV and Beta Herpes Virus Workshop [4]) and can establish persistent infection in the central nervous system in experimental animals with affinity for the limbic system [5]. CMV is found throughout all geographic regions and socioeconomic groups and widely prevalent in the US population [6], [7]. Transmission occurs person-to-person from close interpersonal contact or possibly by contact with fomites [8]. Transmission may also occur through blood transfusion from mother to child through transplacental infection or exposure to breast milk.

Primary CMV infection in immune competent individuals can be asymptomatic or associated with a mononucleosis-like syndrome. However, pre-natal or neonatal exposure to CMV may lead to sensorineural hearing loss, cognitive delay, and permanent neurologic sequelae [9]. CMV also affects immuno-suppressed persons such as those with human immunodeficiency virus (HIV) leading to pneumonia, retinitis, and gastrointestinal disease.

CMV seropositivity has been associated with reduced cognitive functioning [10] and a higher rate of cognitive decline [11] in elderly populations. However, few studies have examined the effect of CMV in non-geriatric adults. A recent study of individuals enrolled in the National Health and Nutrition Examination Survey (NHANES) [12] found that CMV seropositivity was associated with relative cognitive impairment in adults aged 20–59; deficits in the CMV positive group were predominantly found in coding speed and a digit learning task. This study is the only report in the literature examining the relationship between CMV and cognitive functioning in a population of younger adults. In this study, the cognitive battery was limited; in addition, CMV exposure was analyzed categorically as positive or negative, so quantitative associations between antibody level and cognitive impairments could not be evaluated.

In the current study we examined the association between quantitative Cytomegalovirus IgG antibody levels and tests of cognitive functioning in non-elderly adults.

## Material and Methods

The study sample consisted of 521 individuals without a history of psychiatric disorder. Individuals were recruited from posted announcements at local health care facilities and universities in the Baltimore, Maryland area. These individuals were enrolled after they were screened to rule out the presence of a current or past psychiatric disorder with the Structured Clinical Interview for DSM-IV Axis I Disorders, Non-patient Edition [13]. Participants met the following additional criteria: age 18–65; proficient in English; absence of any history of intravenous substance abuse; absence of mental retardation; absence of HIV infection by history; absence of serious medical disorders that would affect cognitive functioning.

The study was approved by the Institutional Review Boards of the Sheppard Pratt Health System and the Johns Hopkins Medical Institutions following established guidelines. All participants provided written informed consent after the study procedures were explained.

All participants were individually administered a cognitive battery, the Repeatable Battery for the Assessment of Neuropsychological Status, Form A (RBANS), [14]. The RBANS consists of 12 tasks which yield scores in 5 domains and a total score. Test indices are Immediate Memory (comprised of List Learning and Story Memory tasks); Visuospatial/Constructional (comprised of Figure Copy and Line Orientation tasks); Language (comprised of Picture Naming and Fluency tasks); Attention (comprised of Digit Span and Coding tasks); and Delayed Memory (comprised of List Recall, Story Recall, Figure Recall, and List Recognition tasks). The index scores are combined to yield a Total RBANS score which is a measure of overall cognitive functioning. Participants were also administered the Wisconsin Card Sorting Test (WCST) [15], [16], 64 card version, a test of set shifting; Trail Making Test part A [17], a test of visual scanning and visual motor speed; and Letter Number Sequencing from the Wechsler Adult Intelligence Scale III [18], a test of working memory. Participants were asked about their educational level and other demographic variables including maternal education as a proxy for socioeconomic status.

A blood sample was obtained at the study visit. We employed solid phase immunoassay techniques to measure IgG class antibodies to Cytomegalovirus in the sera of all participants. Details of the methods have been previously described [19]. Immune reagents were obtained from IBL-America, Minneapolis, Minnesota. Quantitative results were expressed as optical density units based on colorimetric measurements of the immune reactions performed as recommended by the manufacturer. The resulting optical density measurements were normalized to the percentiles of the study population as previously described [20]. To determine if the associations were specific to CMV IgG antibodies, we also measured IgG antibodies to Epstein-Barr Virus (EBV) and Varicella-Zoster Virus (VZV) utilizing the same methods.

### Data Analyses

Linear regression models were used to calculate the association between Cytomegalovirus IgG antibody levels and RBANS Total and index scores, WCST percent perseverative errors, Trail Making Test part A scale score, and Letter Number Sequencing scale score. Logistic regression models were used to calculate the association between low RBANS Total and index scores, defined as < = 80 for the RBANS, in the “borderline” range of cognitive functioning or lower, < = 25^th^ percentile for the Letter Number Sequencing and Trails A scores, and > = 25^th^ percentile for the percent perseverative errors on the Wisconsin Card Sorting Test (because a higher score represents poorer performance), and elevated antibody levels, defined as greater than the 50^th^, 75^th^, and 90th percentiles of the group. All analyses were adjusted for age, gender, race (Caucasian vs. other), maternal education, and participant education. Linear regression methods were also used to calculate the association between Epstein-Barr Virus and Varicella-Zoster Virus IgG antibody levels and RBANS Total score.

All statistical analyses were performed with STATA version 11, College Station, Texas.

## Results

### Characteristics of the sample

The participants had a mean age of 32.8 years (s.d. 11.4, range 19.5–60.6) and 328 (63%) were female. The racial make-up of the sample was 307 (58.9%) Caucasian, 162 (31.1%) African-American, 34 (6.5%) Asian, and 18 (3.5%) other. The mean educational attainment of the sample was 15.6 years (s.d. 2.3) and the mean maternal education was 13.5 years (s.d. 2.9). The mean RBANS total score was 89.4 (s.d. 12.3) and a total of 112 persons (21.5%) had a total score of 80 or lower. A total of 206 (39.5%) of participants were seropositive for CMV. The seroprevalence by race and gender is consistent with that in recent NHANES data [21].

### Association between quantitative levels of CMV antibodies and cognitive scores

Results of the linear regression analyses are depicted in **Table 1**. There was a strong association between the quantitative level of CMV IgG antibodies and RBANS Total Score (p<.0002). There was not a significant association between level of EBV antibodies or VZV antibodies and RBANS Total Score, so no further analyses with these antibodies were performed.

**Table 1:** Association between CMV IgG Antibody Levels and Cognitive Scores (N = 521).

	Coefficient 1	95% CI	p value
RBANS^2^			
Total Score	−1.03	−1.59, −0.46	<.0002
Immediate Memory	−0.58	−1.26, 0.11	n.s.
Visuospatial [27]/Constructional	−1.77	−2.46, −1.07	<5×10^−7^
Delayed Memory	−0.94	−1.48, −0.39	<.001
Language	−0.35	−1.10, 0.41	n.s.
Attention	−0.26	−1.06, 0.55	n.s.
Letter Number Sequencing^3^	−0.15	−0.28, −0.15	<.030
WSCT % perseverative errors^4^	0.16	−0.36, 0.68	n.s.
Trail Making Test A^5^	−0.02	−0.16, 0.12	n.s.

1 Adjusted for age, gender, race, maternal education, and participant education.

2 RBANS =  Repeatable Battery for the Assessment of Neuropsychological Status; total score and index scores.

3 Letter Number Sequencing, scale score.

4 WCST =  Wisconsin Card Sorting Test.

5 Trail Making Test, part A, scale score.

In terms of the association between CMV antibody levels and RBANS index scores, there was also a strong association between levels of CMV antibodies and the Visuospatial/Constructional (p<5×10^−7^) and Delayed Memory (p<.001) index scores of the RBANS. There was also an association between CMV antibody levels and the Letter Number Sequencing Score (p<.03). In all cases, the direction of the association indicated that higher antibody levels were associated with lower levels of cognitive performance adjusted for age, gender, race, maternal education and participant's educational achievement. There were no significant associations between the CMV antibody level and the RBANS Immediate Memory, Attention, or Language index scores or Trail Making Test part A scale score or Wisconsin Card Sorting Test percent perseverative errors (all p>.05).

Covariates also associated with RBANS Total score were older age (coefficient  = 0.23, 95% confidence interval (CI) 0.15, 0.32, p<.001); female gender (coefficient  = 3.24, 95% CI 1.34, 5.13, p = .001); Caucasian (vs. other) race (coefficient 7.79, 95% CI 5.79, 9.79, p<.001); years education (coefficient  = 1.02, 95% CI 0.59, 1.46, p<.001). These same covariates were significantly associated with most of the other cognitive variables in the regression models. Maternal education was not independently associated with RBANS Total or with most of the other cognitive variables.

### Odds of association between low cognitive scores and different levels of elevations of CMV antibodies

**Figure 1:**
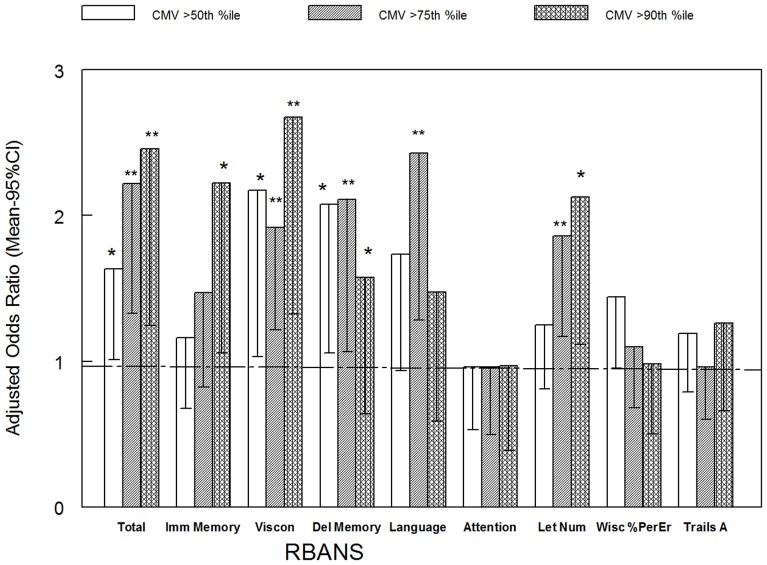
Odds of Low Cognitive Scores by Elevation of CMV Antibody Level. RBANS =  Repeatable Battery for the Assessment of Neuropsychological Status; Imm Memory  =  Immediate Memory; Viscon =  Visuospatial/Constructional; DelMemory  =  Delayed Memory. LetNum  =  Letter Number Sequencing Test. Wisc % Per Er =  Wisconsin Card Sorting Test, % perseverative errors. Trails A =  Trail Making Test, Part A. Adjusted for age, gender, race (Caucasian vs. other), maternal education, and education. *p<.05; **p<.01.

We also employed logistic regression models to determine the odds of association between low cognitive scores, and different levels of elevations of CMV antibodies. Antibodies to CMV were evaluated based on > = 50^th^, > = 75^th^ and > = 90^th^ percentiles. As depicted in **Figure 1**, there was an incremental relationship between the level of CMV antibody elevation and the odds of a low RBANS Total. The odds of a low total cognitive score were 1.63 (95^th^ % CI 1.01, 2.64; p<.045), 2.22 (95^th^ % CI 1.33, 3.70; p<.002), and 2.46 (95^th^ % CI 1.24, 4.86; p<.010) with a CMV antibody level greater than or equal to the 50^th^, 75^th^, and 90^th^ percentile of the group, respectively. Some but not all of the other RBANS scores showed similar quantitative associations. There was also a significant association between the Letter Number Sequencing score and CMV antibodies at the 75^th^ (OR = 1.87, 95% CI 1.17, 2.97) and 90^th^ percentiles (OR = 2.13, 95% CI 1.13, 3.97). There were not significantly increased odds for increased levels of CMV antibodies and the RBANS Attention scores or with the Trails A score or the percent perseverative errors on the Wisconsin Card Sorting Test (all p>.05).

## Discussion

Our study indicates that the level of CMV antibodies is associated with reduced cognitive functioning in a large cohort of non-elderly adults. The association was found when examining CMV antibody measured as a continuous level and also when the CMV antibody was categorized relative to the 50^th^, 75^th^, and 90^th^ percentile level of the study population.

Our findings are consistent with those of Tarter et al [12] in finding an association between CMV seropositivity and reduced cognitive functioning in middle-aged adults. Consistent with the findings of Tarter et al, we found an association between CMV antibody levels and both Immediate and Delayed Memory domains, consisting of both verbal and visual memory tasks. Our study also documents a strong association between CMV antibody levels, measured continuously or relative to specific elevations, for the RBANS Visuospatial/Constructional domain. The tasks in this domain involve figure drawing and judgment of line orientation tasks, both requiring visual spatial abilities. This domain was not measured in the tests performed on the population reported by Tarter et al. Our findings are also consistent with those of Gow et al [10] who examined the effects of CMV seropositivity on cognitive functioning in an elderly population. In this investigation there was a significant association between CMV exposure and lower cognitive ability on memory tasks, as we found in our study, and in overall cognitive score, also consistent with our results in a younger population. The Gow study also did not find an association between CMV seropositivity and performance on speeded processing tasks. In our study, we also did not find any significant association between CMV and performance on speeded processing tasks including RBANS Attention, which includes a speeded coding task, and Trail Making Part A, a test of motor speed. It is of note that the Tarter study did find an association between CMV seropositivity and impaired coding speed using a similar but not identical test. Differences in study populations and in the specific cognitive measures used may account for these discrepancies. It is also of interest that we did not find an association between CMV antibody level and performance on the Wisconsin Card Sorting Test, a measure of set shifting and conceptual thinking; this test was not included in the cognitive batteries of other studies looking at the effects of CMV. Performance on the Wisconsin Card Sorting Test is often associated with prefrontal brain functioning, suggesting that these functions are relatively unaffected by CMV infection. The association between CMV antibody levels and specific areas of brain functioning should be the focus of further investigation.

It is of note that the association between CMV antibody level and reduced cognitive performance was incremental with the degree of elevated antibody level for the RBANS Total score indicating that a higher level of antibody was associated with increased odds of a low cognitive score. The biological reasons for higher levels of CMV IgG antibodies in some individuals are not known with certainty but may be related to more recent exposure to CMV [22], more frequent re-activation of viral infections, strain differences of the infecting CMV [23] or genetic differences in the immune response to infection [24]. Elevated levels of antibodies may also be associated with increased levels of inflammatory markers which in turn mediate changes in cognitive functioning and motor activity [25], [26]. Regardless of the cause, our data indicate that individuals with the highest level of antibodies to CMV are at greatest risk of cognitive impairments.

No association was found between the RBANS total score and either antibodies to Epstein-Barr Virus or Varicella-Zoster Virus, suggesting some specificity of the association with CMV. Previous studies have also documented an association between cognitive functioning and antibodies to Herpes Simplex Virus type 1 [27]. The association between cognitive functioning and other viruses should be the focus of additional studies in larger populations.

Since our study was cross-sectional in nature, we could not identify the timing of primary Cytomegalovirus infection in our population. The finding of Cytomegalovirus antibodies in adults can be the result either of infection occurring around the time of birth or infection acquired during childhood or later in life. Additional longitudinal studies should be performed in order to better define the relationship between the timing of Cytomegalovirus infection and cognitive functioning.

Recently there has been interest in developing new medications for the treatment of Cytomegalovirus associated with chronic conditions [28] and for the prevention of infections through the use of vaccines [29]. The definition of the link between Cytomegalovirus and cognitive functioning might lead to new methods for improving the cognitive trajectory of persons in the general population.
